# Call in the Doctor

**Published:** 1921-06-11

**Authors:** 


### Call in the Doctor.

Shortly after the present coal dispute began, an article which appeared in this section of The Hospital on the relationship of health to industrial unrest attracted a good deal of attention, particularly among medical men. We suggested on that occasion that there is one class of person who is never consulted in these critical industrial discussions, and whose knowledge is hardly ever considered as

**THE PUBLIC WELL-BEING**—(*continued*).

relevant to the free play of industry. That class is the medical class. We have not forgotten the functions of a medical referee, nor do we mean to say that no employer ever consults a doctor on a question which concerns the physical welfare of his employees or on the special conditions operating in an industry abnormally dangerous to health. What we mean was well expressed a few days ago at a London Conference on medical service in industry.

Every big employer who has done any good in the world, said Dr. R. N. Wilson, knows that there is never any profit in underpaying workmen. The healthier the workman, the better his surroundings, the better care taken of him, the more cheerful he is and the greater will be his actual output. "If the doctors show employers and employees that a particular thing—such as bad industrial conditions—is against the interests of both, they may remove the basis of their quarrel and raise industrial disputes to a higher level. . . . The fundamental doctrine of industrial medicine is that there is no disparity of interest between the employer and employee—what helps the one is bound to help the other." These are elementary truths which are only just beginning to dawn not only upon the medical profession, but upon Government authorities, workmen, and employers. We believe it to be literally true that many industrial disputes of recent times could have been greatly modified or prevented altogether if there had been available the means of a scientific analysis of the underlying health issues. We are firmly convinced that on very definite and practical lines there is a great future for industrial medicine in this country.

